# Artisanal salt production in Aveiro/Portugal - an ecofriendly process

**DOI:** 10.1186/1746-1448-7-3

**Published:** 2011-11-04

**Authors:** Carolina M Rodrigues, Ana Bio, Francisco Amat, Natividade Vieira

**Affiliations:** 1 CIMAR/CIIMAR - Centre of Marine and Environmental Research, University of Porto, Portugal, Rua dos Bragas, 289, 4050-123 Porto, Portugal; 2 Instituto de Acuicultura de Torre de la Sal (IATS - CSIC), 12595 Ribera de Cabanes (Castellón), Spain; 3 Department of Biology, Faculty of Sciences, University of Porto, Portugal. Rua do Campo Alegre s/n, 4169-007 Porto, Portugal

**Keywords:** Salt production, Salinas, Coastal wetlands, Biodiversity, Ecosystems, Aveiro, Portugal

## Abstract

Solar salinas are man-made systems exploited for the extraction of salt, by solar and wind evaporation of seawater. Salt production achieved by traditional methods is associated with landscapes and environmental and patrimonial values generated throughout history. Since the mid-twentieth century, this activity has been facing a marked decline in Portugal, with most salinas either abandoned or subjected to destruction, making it necessary to find a strategy to reverse this trend.

It is, however, possible to generate revenue from salinas at several levels, not merely in terms of good quality salt production, but also by obtaining other products that can be commercialized, or by exploring their potential for tourism, and as research facilities, among others. Furthermore, with an adequate management, biodiversity can be restored to abandoned salinas, which constitute important feeding and breeding grounds for resident and migratory aquatic birds, many of which are protected by European Community Directives.

The aims of this manuscript are to present a brief overview on the current state of sea salt exploitation in Portugal and to stress the importance of recovering these salinas for the conservation of this particular environment, for the regional economy, the scientific community and the general public. The Aveiro salina complex is presented in detail, to exemplify salina structure and functioning, as well as current problems and potential solutions for artisanal salinas.

## Brief historical background of salt exploitation in Portugal

Portugal, with its extensive coastline exposed to hot and dry winds and constantly high temperatures during summer, has always shown favourable conditions for the development of salinas that use the renewable eolic and solar energies to produce salt. The country has a privileged geographical situation for salt production, in comparison to other European and even non-European countries [1]. In Portugal, salt production through solar evaporation of sea water was introduced by the Phoenicians in the 9^th ^century BC [2] and, during the Roman period, salt must have been intensively exploited, as there are abundant archaeological remains of fish salting settlements in several places in southern Portugal. In the beginning of the 10^th ^century, the exploitation of salt, along the northern coastline, between the Minho and Vouga rivers, was already prospering [3]. In the 12^th ^century, even before the Portuguese maritime expansion, Portuguese salt was regarded as a high-quality product in various parts of the world [4]. Salt exploitation was then of utmost importance to the national economy [5] and the Ria de Aveiro, a natural lagoon at the mouth of the Vouga river, became particularly important, providing, by 1178, enough salt for the whole country and for large exports abroad. The increase of salt-related activities in the region of Aveiro resulted in the decline (and in some cases, particularly during the 14^th ^and 15^th ^centuries, the complete disappearance) of other salt producing centres, to the north of the Douro river, whereas areas to the south of the Ria de Aveiro continued to prosper [3]. There is documentary information about salinas existing in practically all Portuguese Atlantic coast estuaries and lagoons, with references to salinas in the northern estuaries of the rivers Minho (near the town Caminha), Cávado (Esposende), Ave (Vila do Conde) and Douro (Porto), and the bay of São Martinho do Porto (Salir do Porto), which however disappeared for economic or coastal dynamic reasons, leaving no physical traces by the end of the 20^th ^century [6,7]. In that century the artisanal salt production activity consolidated in its original regions: the Ria de Aveiro and regions south of it, with salinas in Figueira da Foz, the Óbidos lagoon, in estuaries of the rivers Tagus, Sado and Mira, and in the Algarve. The exploitation of salt springs (inland salinas) was, until the early 20^th ^century, confined to Rio Maior. In the first half of that century, further salt springs were discovered in the region of Leiria, which have also been exploited [7].

After 1936, the salt industry entered a deep crisis due to the development of cold conservation techniques and the appearance of industrialized salt-winning processes that could provide higher salt production at lower cost. This situation led to a progressive decay of artisanal salt-production; a decay that peaked in the 1980s, when Portugal became an EEC/EU member and subsidies were attributed to agriculture and aquaculture, encouraging people to exchange the seasonal salt production activity for an alternative continuous activity. The roughness of the work itself, not compensated by the revenues obtained and the lack of incentives given to continue this activity, further contributed to salina abandonment [8-11].

In the last 20 years, Portuguese salt-culture suffered a further decline with a reduction of more than 50% in active (i.e. salt producing) salinas, generally resulting from the transformation or complete abandonment of salinas, leaving them exposed to destruction. Currently active salinas are still found at the southern Portuguese coast (Faro, Olhão, Castro Marim and Tavira) and in the central and southern parts of the Atlantic coast, in the estuaries of the Sado (Setúbal), Tagus (Alcochete), Mondego (Figueira da Foz) and Vouga (Aveiro) rivers, with inland salinas being still exploited in Rio Maior. With the decline in the production of artisanal salt, associated environmental, cultural, historic and human values are lost, as artisanal salinas maintain a high biodiversity, constitute a highly valued man made landscape and are part of our industrial heritage [12].

## Artisanal salt production in Aveiro

### Geographical and historical background

**Figure 1:**
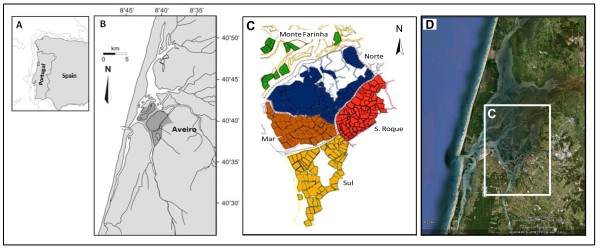
**Aveiro's salina complex**. (A) Location of Aveiro's salina complex in Portugal; (B) The Ria de Aveiro lagoon and the location of the salina complex (shaded area) [11]; (C) The five groups of Aveiro's salina complex [*adapted from *[11]]; (D) Aerial image of the salina complex (box) [*source: Google Earth, 2010*].

The Ria de Aveiro is a lagoon system located at the northwest Atlantic coast of Portugal (40°38'N, 8° 44'W) (Figure 1). It has an approximate length of 45 to 47 km (NNE-SSW) and a maximum width of about 10 to 15 km. The lagoon is influenced by tides and displays vast salt marches, providing excellent conditions for the development of diverse characteristic biotopes and for the construction of salinas [13]. It is therefore not surprising that salt production became one of the earliest and most important industrial activities in the region.

The Aveiro salina complex located within the Ria de Aveiro, occupies an area of about 2, 600 ha, including productive and non-productive areas. This complex is organized into five groups separated by main channels: Monte Farinha, S. Roque or Esgueira, Norte, Mar and Sul (Figure 1C) [9,14].

The first known document related to salt production in Portugal (the testament of the Countess Mumadona) dates back to 929 AD, which is before the formation of the nation, and refers to the donation of one salina in Aveiro [15]. Over the centuries, Aveiro's salinas have varied in number and production, alternating between periods of decadence and others more favourable to production. Until the 17^th ^century, salt from Aveiro was not only commercialized in the entire country but also in Europe (especially in the north), being essentially used to salt meat and fish, namely cod. During this period, Aveiro offered the best conditions for the construction of salinas, due to its climate and type of coast. And salt producers successfully responded to the needs of the growing local population and the Portuguese economy in general, turning Aveiro into the national salt production centre, both in terms of salt quantity and quality.

However, during the 17^th ^and 18^th ^centuries, salt production in Aveiro declined markedly due to lagoon inlet problems and the (close to) stagnation of the lagoon water. The varying topography of the Ria de Aveiro and the associated water circulation patterns constituted thus decisive factors for the number and production of the salinas in Aveiro [16]. In 1808 a new (artificial) lagoon inlet was inaugurated and in 1815 an artificial channel was built, since the natural river bed of the Vouga was not deep enough to drain major floods, which destroyed the salinas' protection walls and excavated their bottoms, contributing to salina degradation and abandonment. Consequently, the number of salinas in Aveiro, which reached 500 in the 10^th ^century, decreased to 170 in the 17^th ^century. The hydraulic constructions of the 19^th ^century established adequate conditions for salt production, increasing the number of salinas and restoring the region's salt-producing economy till the mid-20^th ^century [16,17].

In 1970, approximately 270 salinas were active in the lagoon, covering 1661 ha and producing an average of 60, 000 tons of salt per year [17]. Since then, salt production went into decline. Many salinas were abandoned and converted into aquaculture facilities, mostly because of the lack of profitability due to the economic crisis affecting the artisanal salt sector. Reasons for this shift were not only the opening of markets, technological changes in fishing and industry, and the lack of salt workers, but also the state of degradation of the Ria de Aveiro (with high pollution, increasing motorised navigation and consequent increase in the ripple of waters and the destruction of pond walls, among other causes). The collapse of the cod fishery, which was a major consumer of salt from Aveiro, and the silting up and degradation of the Ria's channels (causing increased difficulties in transporting materials for the reconstruction of the walls, as well as in transporting the salt for storage), have contributed to the aggravation of circumstances. All these factors have led to a significant increase in production costs and decrease in revenues that did not allow for compensation for the inherent work or returns on the invested capital [8,16-18].

Data for 2007 indicate that only 3.3% of Aveiro's salinas were still used as active or semi-active salinas, 5.2% were inactive salinas, 16.1% occupied with aquaculture and 72% completely abandoned [11], which attests to the decay of salt-culture. Present trends suggest that the complete abandonment of artisanal salt production may be eminent, which will seriously affect ecological balance and quality of the landscape [9]. For the year 2009, there is information about nine active salinas in the Aveiro complex (Podre, Santiago da Fonte, Pajota, Troncalhada, Senitra, Grã-Caravela, Passã, 18 do Caramonetes and Puxadoiros) dedicated to artisanal salt production, producing 908 tons of salt (which constitutes an increase of about 44% in comparison to 2008) [14]. By 2011 the salina Podre had shut down, leaving only eight active salinas (pers. communication of Margarida Ribeiro, Aveiro Municipality). It is however remarkable that, despite its practically irrelevant importance for the regional economy, this activity has been maintained and salt is still produced in the same ancient way, by hand and with several crops per salting season [19].

### Salina structure

Solar salinas are man-made systems for the extraction of salt from seawater, by means of solar and wind evaporation. Structurally, they are a connected series of shallow ponds through which seawater flows and evaporates, delivering brine nearly saturated with sodium chloride to crystallizer ponds where salt is deposited [20-22]. Traditional Portuguese solar salinas can be grouped into four typologies, each corresponding to a different region and exploitation method: the Algarve, the Sado, the Tagus, and the Figueira da Foz and Aveiro types. There are, however, similar basic conditions for all of these Portuguese typologies: an average high-tide level of 3 to 3.5 m, with spring tides going as high as 4.8 m, an average salinity ranging from 35 to 300 ppt and prevailing NW winds in summer. And, although these salinas are largely protected from erosion caused by ocean waves, they are still affected by the erosion resulting from water circulation inside the estuarine systems, particularly during floods that coincide with spring tides [7,19].

Aveiro and Figueira da Foz salinas can be assigned to the same category because, although they follow different nomenclature and trace, they do share many common characteristics: they are small units (usually not larger than 10 ha), have numerous compartments (7 to 9), the harvesting of salt occurs at intervals of 3 days and much of the salina's surface (about 40 to 50%) is occupied by the water reservoirs [7,19].

**Figure 2:**
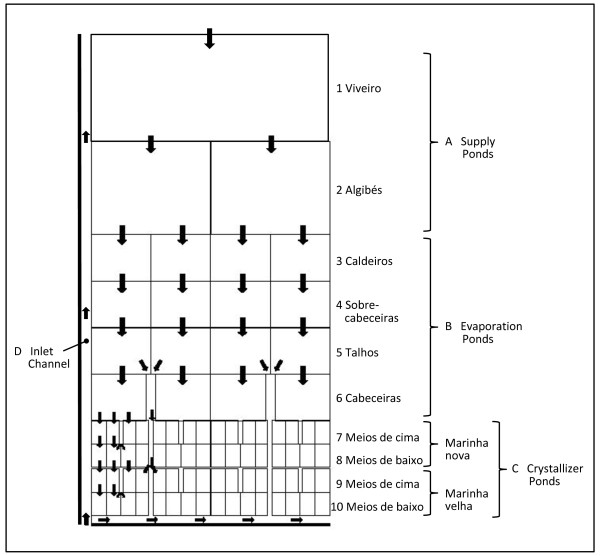
**Scheme of a typical salina in Aveiro (with local terms for the different pond types)**. A: supply ponds; B: evaporation ponds; C: Crystallizer ponds; D: inlet channel (the direction of the water flow in the various compartments is indicated by arrows; in the crystallizer section flows are only indicated for some ponds) [*adapted from *[18]].

Following the inlet channel, there are basically three types of compartments in an artisanal salina: supply ponds (designated in Aveiro as "viveiro" and "algibés"), evaporation ponds (which are arranged in "caldeiros", "sobre-cabeceiras", "talhos" and "cabeceiras", depending on their position relatively to the supply ponds) and crystallizer ponds. Like in salinas of some other European countries (e.g. France, North of Spain), the crystallizer ponds in Aveiro are subdivided creating an extra type of compartment - the condenser ponds. These are used to obtain purer sodium chloride, through repeated water exchanges between these and the crystallizers [11]. In Aveiro, the crystallizer ponds account for about 10% of the total area of the salina and are composed of condensers (or "meios de cima") and crystallizers (or "meios de baixo") distributed in two series ("marinha nova" and "marinha velha") (Figure 2).

The supply ponds are the biggest and deepest of the system (Figure 2A). They communicate with the sea through an inlet channel which supplies seawater to the system during spring tides. The evaporation ponds (B) constitute the largest evaporation surface. They receive water from the supply ponds and supply brine to the crystallizer ponds (C) where the deposition of sodium chloride takes place. Communication between ponds takes place digging gullies into the ground, through openings in the dyke closed with wooden structures (sluices), or through circular openings that are closed with mud.

Although solar salinas worldwide use seawater of similar chemical composition as the raw material for salt production, the size and quality of the halite crystals that precipitate in the crystallizer ponds are highly variable [23]. Sodium chloride produced from seawater is the chief or only product of most salinas, but potassium sulphate, potassium chloride, sodium carbonate and other salts may be obtained and manufactured [24]. The quality of salt (sodium chloride) is determined not only by the characteristics of salt crystals (their size, hollow or solid structure), but also by the concentration of contaminants (calcium, magnesium, sulphate and insoluble matter) in the salt. Seawater contains a certain amount of dissolved salts, mostly sodium chloride, but also bromides, carbonates, calcium and potassium salts, which give salt an unwelcome taste when precipitating simultaneously. Salt quality depends therefore on the differential precipitation of the diverse salts present in the brine [25,26]. Crystals should be large, single, solid and transparent, with contaminants not exceeding 0.03% to 0.05% of calcium, 0.02% to 0.04% of magnesium, 0.11% to 0.16% of sulphate, and 0.01% to 0.02% of insoluble matter [22,25]. This is the reason why the salina is composed of a series of different compartments, which communicate with each other through water gates that control brine flow. As the brine goes from one reservoir category to the next, the height of water steadily decreases and the relative surface of exposure gradually increases, aiding evaporation and resulting in a salinity gradient between the first and the last compartment. Because each salt precipitates at a given salinity, the different salts will precipitate fractionally in the different compartments [16,26].

The construction of coastal salinas depends on certain conditions: i) they must be close to the sea, close enough for the effect of the tides to be felt; ii) they must be located in a flat region practically at sea level, allowing them to be shallow and wide, iii) they should be located in estuarine areas, where the soil, though sandy, contains clay that gives a certain impermeability to the floor and iv) they must be located in climatic regions with seasons when overall evaporation exceeds overall precipitation [27].

The sediment of the Ria de Aveiro lagoon is mostly composed of silty sand and loamy silt, with 20 to 90% of silt-clay [28]. This grain size distribution is very important for artisanal salt production, because sediments avoid infiltration of fresh ground water into the salt ponds, which would hamper the crystallization of the salt.

The amount of sodium chloride annually produced by a solar salina is variable, mainly depending on atmospheric conditions for evaporation such as air temperature, wind intensity, atmospheric humidity and water exposure surface. Evaporation is faster when temperature, wind intensity and the exposed water surface are higher. And evaporation becomes slower with higher atmospheric humidity. Since the winds have a huge influence on the speed of evaporation, the orientation of the compartments is of critical importance. For a more effective action of the winds, pond orientation should allow predominant winds to blow diagonally over the compartments, from the deepest to the shallowest ponds. Notice that the Aveiro region is influenced by the Atlantic Ocean, which means that the weather conditions here are less favourable than those in more Mediterranean-type climates; on average it rains more often and more heavily in summer and the solar irradiance is considerably lower, leading to lower salt productivity per hectare [7].

## Artisanal salt production process

### Physicochemical system

Seasonal salinas fallow during winter and produce salt only during the summer, when climate conditions are favourable. They are found in climates with warm, dry summers, and where 65 to 80% of the annual rainfall occurs during winter months (e.g., shores of Atlantic Europe, the Black Sea and Mediterranean Sea, Namibia, South Africa, Yellow Sea, San Francisco Bay) [20,25]. For Portuguese salinas, salt production is restricted to a period, from spring to autumn [16]. After this period, the sluices are opened during high tide, and the salina is flooded. It stays flooded until March or April, when temperatures rise and atmospheric precipitation decreases [11]. In the non-productive season, the salinity in the whole system approaches that of the supply water, i.e. ≤ 35 ppt, while water depths vary per compartment. During this season water depth in the supply ponds, evaporation ponds and crystallizers is approximately 70 cm, 80 cm and 100 cm, respectively [29]. When the productive season begins, the ponds are emptied and prepared for a new cycle of salt production [11]. It is necessary to proceed with specific works on the salt ponds bottoms, which include the reparation of the separation dikes, replacement of damaged woodwork and preparation of the tools for salt production. Once all these works are concluded, it is necessary to clean the crystallization basins. Well-maintained and carefully prepared basins are essential for the production of high quality salt [19]. In June, the ponds are filled again. They are subsequently supplied with new sea water every 15 days, during spring tides. A slight level gradient between the supply and crystallizer ponds provides a water flow by gravity [30]. The passage of water must be regulated and timed, so that it flows into each following tank more concentrated, reaching saturation in sodium chloride on arrival at the crystallizers. During the productive season, water depths in the supply, evaporation and crystallizer ponds is about 20 cm to 100 cm, 10 cm to 50 cm, and a few centimetres, respectively. The salinity is generally below 70 ppt in the supply ponds, varies between 70 ppt and 290 ppt in the evaporators and is very high in the crystallizer ponds, reaching more than 300 ppt. It is in the evaporation ponds that seawater is concentrated up to the saturation point of NaCl, and where the various salts crystallize which are less soluble than NaCl, such as Fe_2_O_3_, CaCO_3_, BrO_3 _and CaSO_4_, which precipitate at salinities of approximately 95 ppt, 95 ppt, 130 ppt and 160 ppt, respectively [11,29]. The high surface-to-volume ratio in the ponds promotes evaporation as the brine slowly flows to each succeeding, more saline pond in the series. The aim of this form of brine management is to allow precipitation of the less soluble marine minerals ahead of the crystallizers, so that only NaCl precipitates in these ponds [31].

**Figure 3:**
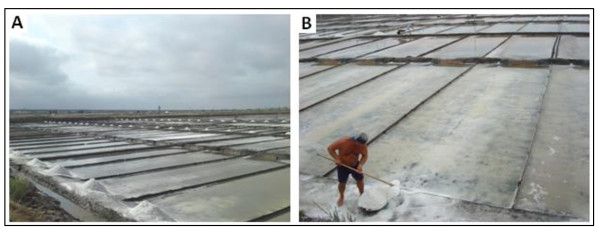
**Salt production in the Troncalhada salina, in Aveiro**. (A) Extracted salt drying next to the crystallizers; (B) Salt harvest in the crystallizers.

When the water volume is reduced to 1/10 of its initial volume in the evaporation ponds, the brine flows into the crystallizers. There it stays until reaching 1/40 of its volume in order to obtain the precipitation of NaCl on the bottom at 220 ppt, followed by the more soluble salts in the brine, such as MgSO_4 _(260 ppt), MgCl_2 _(275 ppt) NaBr (284 ppt) or KCl (294 ppt) [11,29]. The supernatant liquid (bittern), rich in compounds of magnesium, potassium chlorides and sulphates, is removed and the deposited salt (the crop) is harvested [25]. In the salinas of Aveiro, salt (NaCl) harvesting (Figure 3) takes place in the crystallizers ("meios de baixo") and only exceptionally (i.e. in very hot summers) in the condensers ("meios de cima"), originally created to obtain purer sodium chloride.

The salt deposit on the floor of the crystallizers is very consistent and compact. During the harvest this salt has to be released, broken and washed (with the crystallizer brine). Some care has to be taken not to mix the salt with the sediment. The harvested salt is piled up on wooden boards next to the crystallizer pond, and left to dry. When thoroughly dried the salt is stored, after removal of the upper layer of the heap, which has become dirty due to its exposition to dust (this salt is returned to the crystallizer ponds to contribute to the following harvest). During the warm months salt production is continuous. At Aveiro, the crystallizers are supplied with brine at 3-day intervals.

Some artisanal salinas, including those in Aveiro, also produce *fleur de sel*; a salt rich in minerals, with a particular taste and high market value. As the water volume is reduced to the required fraction (1/40) of the supplied sea water, the *fleur de sel*, which consists of sodium chloride and traces of different salts, begins to form on the crystallizer ponds' water surface. This floating salt is collected once or even twice a day (except when it does not form due to heavy winds or when humidity is high). *Fleur de sel *does not undergo any form of processing. It is directly packaged and commercialized.

### Importance of physicochemical and biological systems in salt production

Salinas are mostly closed systems, exposed to annual drainage and drying. Contrary to natural brackish environments (lagoons, estuaries and inland seas) and as a result of the annual cycle of salt production, seasonal salinas present two ecologically distinct periods (production and non-production) with distinct physicochemical and biological conditions. During the non-productive season, when the salinas are flooded, environmental factors do not show important variations and the systems are more stable for living organisms [11], holding high fauna and flora diversity, distributed over all of the ponds, with the deeper supply ponds showing the highest diversity of organisms. During the salt production season, characteristic biota, adapted to different salt concentrations, inhabit the salina ponds along the salinity gradient [32]. The increase of the proportion of salts dissolved in the brine causes a reduction in the specific diversity, with less-eurihaline organisms being eliminated [26].

The living organisms in the brine of a salina constitute a biological system or ecosystem essential to the salt production process, which is intimately linked to the system's physicochemical phenomena [24,33]. The exchanges with the sea, the hydrology, salinity and nutrients play an important role in the development of the biological communities [34]. Depending on its type and development, the ecosystem's performance is often responsible for the degree of success (i.e. product quality and output) of a salina [33].

The aim of every solar saltwork or salina with seawater intake is the achievement of a continuous and economic production of high quality salt (sodium chloride). One way to maintain or increase the salt production in the Aveiro salinas and to improve its quality is to simultaneously manage and coordinate the physicochemical and the biological systems in the salina ponds, from the inlet channel to the crystallizer ponds [31,35]. Therefore, continuous collection, display and utilization of information are required, which indicate the physicochemical and biological status of the salt field, efficiency of the harvest and wash processes, and concentrations of critical ions and insolubles in the washed salt. Such information allows manipulation of control features, to make routine adjustments, anticipate and obviate developing problems, and enable optimum and harmonious performance of the physical and biological systems [24].

### "Balanced", "inadequate" and "unbalanced" biological systems

The salina's biological system, composed mainly of microscopic organisms suspended in the water (the planktonic community) and attached to ponds floor (benthic communities forming mats), can aid or harm the salt production [24]. Knowledge of the ecology of these communities is therefore of utmost importance for salt production. Each community consists of: producer organisms (algae, cyanobacteria, and certain bacteria) that manufacture organic substances from light energy, carbon dioxide, and inorganic nutrients (through photosynthesis) and power the entire biological system; and, consumer organisms (*Artemia*, brine flies, bacteria, ciliates, crustaceans, molluscs, nematodes), which use organic substances to power their physical activities, growth and reproduction [34].

Three types of biological systems with different characteristics can be considered in salinas: "balanced", "inadequate" and "out of balance" biological systems. It is well known that a "balanced" biological system in salt ponds is essential for salt production, whereas an "inadequate" or "unbalanced" system creates problems for salt precipitation [33,36]. A "balanced" biological system produces optimum quantities of organic materials properly distributed between plankton and bottom communities living in a salina, promoting the production of high quality salt. The planktonic community of such a system (which includes algae, bacteria, protozoa, and brine animals suspended in the brine) contributes to salt production, colouring the brine and increasing solar energy absorption and water evaporation [33,37]. The benthic community (which consists of microorganisms, small molluscs and nematodes growing on the floor of the ponds as mats and deposits) favours salt production promoting the development and maintenance of mats firmly attached to the pond floor, which sustain the desired thicknesses, preserve biodiversity, remove and permanently retain nutrients from the overlying water, seal ponds against brine leakage and fresh groundwater infiltration, and help prevent dominance of undesirable mucilage producers [20,33,34].

An "inadequate" biological system lacks sufficient organic productivity; salinas with these systems experience insufficient brine colouring and evaporation, and leaking ponds. An "unbalanced" biological system, caused by a sub-optimal distribution of organic materials between the planktonic and benthic communities, can result in mucilaginous brine, decreased evaporation, and a low-quality product (particularly in terms of colour, insolubles and sulphate content, and crystal size of the salt) [33].

Even when salinas have functioned successfully for a number of years, their biological systems are sensitive to internal and external ecological changes that can lead to serious problems [33]. Microbiological problems in the salinas (e.g. poor mat development and slime production) may be anticipated based on a regular monitoring program of nutrient analyses, plankton and mat evaluations [31]. Hence, knowledge about the variability of the ecological factors is necessary to be able to maintain or increase the salt production and improve its quality, proceeding with a careful biomanipulation of the system, when necessary.

### Biological spatial organization in salina ponds with a "balanced" biological system

During the salt production season, a biological spatial organization consisting of ecological entities that can be relatively autonomous and stable forms along the salinity gradient, in the consecutive salina compartments. The formation of such biological entities along the gradient, which ranges from seawater in the supply ponds to extreme hypersaline environments in the crystallizing ponds, gives salinas a high eco-physiological and ecological value [35]. From low to high salinities: 1) the benthic community changes from loosely organized mats of many species to leathery mats of several layers dominated by fewer species; 2) the composition of planktonic community gradually changes from low levels of diverse groups of species (kinds) to high concentrations of few important species; 3) photosynthetic production of new organic substances that exceed consumption gradually changes to consumption surpassing production; and 4) concentrations of dissolved and particulate organic substances increase in the water [34]. The later increase is due to brine concentration by evaporation and due to the inability of bacteria in the downstream ponds to breakdown organic matter as fast as it arrives. Salinity increase also introduces a succession of organisms that allow reuse of essential minerals and organic matter. Thus, when organisms flow from lower to higher-salinity ponds, they die making their minerals and organic matter available to a new set of organisms [33].

The supply ponds display the greatest organism diversity, biological productivity of organic matter and ecological stability of the entire system [33]. In these ponds the variety and concentration of microorganisms suspended in the water column are similar to the plankton of the nearby marine environment. Seagrasses, seaweeds, fish, and other large marine life are also well represented in the supply ponds [24].

In the evaporation ponds, where salinity during the production season is 3 to 7 times higher than that of sea water, specific diversity decreases though the density of some species may be slightly higher [27]. Specialized biota develop in these ponds, but most noteworthy is the plankton component consisting of cyanobacteria, brine flies and brine shrimp. In a "balanced" biological system, non mucilage producing planktonic cyanobacteria thrive, conferring dark colours to the brine. In a salina whose ecosystem is out of balance, often caused by excessive nutrients and severe brine dilution, the planktonic cyanobacteria *Aphanothece halophytica *may predominate and harm the salina [25,33]. Disturbances cause *A. halophytica *to reproduce at high rates, excluding competing species (e.g. *Artemia *sp.) and secreting abundant polysaccharide slime [34,38]. When this polysaccharide material reaches the crystallizer ponds downstream, this may result in an increase in brine viscosity and the production of soft, poor-quality salt crystals [38]. In the crystallizers, effects of high viscosity include: decreased effectiveness of the red halophilic Archaea; crops or salt floors unable to support machinery (in industrial salterns); harvests of small and hollow crystals that bind or retain dissolved and crystalline contaminants; and, increased development of *Dunaliella salina *populations [24,38]. These green algae are generally of great importance, being part of the diet of *Artemia *and contributing to the increase of solar radiation absorption. Disturbances, however, may cause *D. salina *to reproduce massively, and release damaging quantities of organic substances that are highly detrimental to salt production [24,25,34].

Depending on the intake water of the salina, brine fly larvae and brine shrimp feed on organisms and debris produced in the low salinity ponds [33]. Brine shrimp *Artemia *thrive in the highly saline evaporation ponds, but their numbers and activity decline sharply in the crystallizers due to a variety of environmental factors (e.g., the metabolic cost of osmoregulation in strong brines, elevated brine temperatures and low oxygen levels) [25,31]. Brine shrimp living in the evaporation ponds synthesize more haemoglobin, expend greater amounts of energy and thus consume more food than they would require at lower salinities, because of the extra osmoregulation effort needed for survival. This "forced" feeding by brine shrimp is of particular value to solar salinas [33]. Brine shrimp ingest suspended calcium sulphate (gypsum) crystals, and efficiently utilize much of the microplankton and other organic substances they consume. These shrimps deposit their wastes in membrane-bound faecal pellets that fall to the floor and become incorporated into the benthic community. These activities clear the water, aid maintenance of the mat, prevent recycling of nutrients, control phytoplanktonic *Aphanothece halophytica *and *Dunaliella salina *populations, and assist the delivery of high-quality, nutrient-depleted brine, to the downstream ponds. *Artemia *may ingest but not utilize *A. halophytica *but ingest and utilize *D. salina *[24,25].

In the crystallizers, the salinity reaches values exceeding the tolerance limits of *Artemia *and brine fly larvae. These organisms die, sink and stay deposited at the bottom in large quantities, constituting the main protein source for the resident red halophilic Archaea which are the key biological component of the crystallizer ponds [22,33,39,40]. Red halophilic Archaea are heterotrophs that thrive on the dissolved organic carbon, which passively increases by concentration as the water flows downstream to the crystallizers [32]. In these ponds the few species that exist are often present in high concentrations [41]. The plankton, mostly several genera of red halophilic Archaea (*Halobacteriaceae*), reaching or exceeding 10^8 ^cells ml^-1^, feed on dissolved organic substances and colour the brine pink to bright red because they contain 50-carbon bacterioruberin carotenoids in their cell membrane, and sometimes also retinal proteins (bacteriorhodopsin, halorhodopsin) [23,24]. By consuming significant quantities of organic substances, and by absorbing heat energy from the sun, high concentrations of the red halophilic Archaea decrease organic substances in the brine, improve evaporation, increase the quality of the salt, and improve crystal characteristics [24]. When excessively high concentrations of nutrients reach the evaporation and crystallizer ponds, *Dunaliella salina*, rich in β-carotene, may form populations sufficiently dense to colour the brine red-orange [20]. It was recently recognized that a rod-shaped, extremely halophilic representative bacterium of the phylum *Bacteroidetes *(*Salinibacter*) may also be present in significant numbers in those environments in which red halophilic Archaea thrive, such as the crystallizer ponds. This bacterium contributes to the brine coloration (orange-red) due to the presence of the pigment salinixanthin (an unusual acylated C40- carotenoid glucoside). The carotenoids of the bacterioruberin group of the members of the family Halobacteriaceae appear however to be the main factor causing the characteristic red colour of hypersaline brines worldwide [23].

## Salinas' ecological and conservation values

Through the centuries, man has developed and refined the techniques of salt making, creating many different types of salinas, well adapted to the various local conditions [19]. Salt production in Portugal is still carried out in a traditional manner, from salt pan cleaning to salt harvest, transportation and storage, with saltworkers responsible for the organisation and carrying out of the several heavy tasks from spring to autumn [17]. Artisanal salt production is therefore an ancient activity, which has become a completely integrated part of saline ecosystems. Salinas can be considered as particular ecosystems, where human intervention is tolerated and even necessary to maintain its characteristics, preserving the landscape and protecting the environment, while simultaneously generating economical profit [42]. Salinas are thus integrated ecosystems that can effectively produce an economically viable product while serving a critical role in nature conservation and biodiversity [43].

Salinas form complex ecosystems and help sustaining diverse live forms including microorganisms and macroflora and -fauna, such as adapted vegetation, marine, avian and terrestrial fauna. Environmental conditions in the salinas - especially their seasonally varying hydrology and hypersalinity - limit the occurrence of many plant species. Vegetation is therefore poor in terms of biodiversity, but increases the local ecological value [44]. The vegetation that manages to survive under these conditions is generally of small size and has morphological adaptations as well as physiological mechanisms for osmoregulation. These plants have salt glands to eliminate salt and avoid the uptake of high salt concentrations. The vegetation observed in salinas from Aveiro is mainly composed of *Salicornia ramosissima*, *Sarcocornia perennis*, *Halimione portucaloides*, *Tamarix africana*, *Juncus maritimus *and *Spartina maritima *[45]. Various species of *Salicornia *have been used for human consumption, in salads or as a "green" salt substitute. There are also studies suggesting that some *Salicornia *species have medicinal properties (e.g. immunomodulatory, antioxidative, anti-inflammatory, anti-hyperlipidemic, and antidiabetic properties) [46]. The ecophysiology of *Salicornia ramosissima*, a species that is commonly found in channel margins and salinas in Aveiro, has been studied to optimize its production and allow for a sustained exploration of regional salina vegetation resources, for both food and medicine [47].

The conservation value of a salina wetland is different from that of a freshwater wetland, but the biomass and species diversity in a salina wetland can be just as large and important as in a freshwater wetland [48]. In Portugal there is no specific legislation for the conservation of wetlands, however the majority of its coastal wetlands are legally protected, either as Nature Reserves or Natural Parks. All of these nationally classified areas were also designated as Special Protection Areas (SPA's) within the frame of the Bird's Directive (Dir. 79/409-CEE) [19]. When evaluating the natural value of wetlands for conservation as protected areas, bird species are the ones most frequently considered. Abundance, presence, or absence of birds have proven to be effective indicators of biological integrity in wetlands and are also considered to be good indicators of a site's ecological condition and of environmental changes therein [49]. The Ria de Aveiro, where the Aveiro salina complex is located, is one of the most important ecological coastal wetland areas in Portugal [50], particularly for the conservation of resident and migratory aquatic birds, functioning as a breeding and feeding site for a large number birds [51]. Application of the EU Bird Directives gave this lagoon the Special Protection Zone (PTZPE0004) status, with inclusion in the Natura 2000 Network.

Given their particular characteristics, such as the fact that they are less affected by tidal variations, the salinas in the Ria de Aveiro lagoon are regularly occupied by more than 20, 000 birds, mainly waterbirds, some of them protected by European Directives [42,52]. Species that prefer to nest in salinas include: the Pied Avocet *Recurvirostra avosetta*, the Blackwinged *Himantopus himantopus*, the Little Tern *Sterna albifrons *and the Kentish Plover *Charadrius alexandrinus*; species that are not only particularly interesting but also belong to the Species of European Conservation Concern (SPEC), being SPEC 3 category, which corresponds to species with an unfavourable conservation status, as well as being listed in the Bern (Annex II) and Bona (Annex II) conventions and the "Bird" directive (Annex I) [51].

In many estuaries and South-European coasts, salinas are the most important man-made habitats for birds [6]. In winter, migratory birds - ducks, geese, waders, birds of prey - from northern Europe stop by or spend several months in Mediterranean and Atlantic salinas. These manmade wetlands are thus also of great interest because their ecological function exceeds their boundaries and integrates an important global network of wetland systems [49]. Aquatic and migratory birds that inhabit or pass by an estuary face the specific problem of obtaining enough food for their maintenance and/or enough fat accumulation for the continuation of their migratory journey. Since most are incapable to eat whilst swimming, feeding is limited to a period of 7.5 to 8.5 hours, the periods of low tide [53]. A great number of biotic factors, such as high concentration of birds and the fluctuation in prey population effectives, and abiotic factors, namely the short duration of days, low temperatures and adverse meteorological conditions, acting together or separately, may reduce even further the potential rate of food consumption [54]. Salinas, although partly conditioned by the local management system, are less affected by tides, maintaining water levels that allow birds to extend their feeding periods [55]. From a conservationist point of view, these habitats constitute thus valuable alternative or additional feeding areas that may become crucial when the normally used feeding grounds are not available or their use is conditioned. Furthermore, birds (among other important groups of organisms) take advantage of the extensive gradient of physicochemical conditions, and consequently the wide range of food resources from which they extract, according to their energetic needs, the most lucrative items. *Artemia *adults and cysts are the principal food source for many waterbirds in salinas. The birds, on the other hand, contribute indirectly to an increased salt production, fertilizing the ponds with their faeces and increasing the growth of phytoplankton [11,55].

In order to be attractive for the birds, salinas require an active ecological management. The general aim of an ecological management is to provide the birds with food, protection and suitable nesting sites. It is partly a question of managing the water levels to prevent the nests from being flooded and the pools from drying out. Artificial regulation of the water levels has to be done without interfering with the production of salt. The water has to be supplied to the extraction area of the salina with the necessary salt concentration and at intervals that are adequate for a normal exploitation. However, during the breeding season, a more stable water level will have to be maintained in the initial compartments, to create stability and a more predictable environment for the birds. The maintenance of more stable water levels in those areas of the salinas will also allow for a more efficient control of ground predators [19].

## Salina abandonment and its consequences

The future occupation of the Aveiro salinas is of special concern, as the decline in artisanal salt production has led to increased salina abandonment, with a consequent loss of cultural, landscape and ecological values. Traditional salt production has ethnological values associated to salina architecture, the traditional ways of production and equipment used, working and social conditions of the people employed in salt making, and even the product itself as a cultural element [19,56].

The lack of maintenance work in abandoned salinas has also led to dyke erosion, causing silting up of ponds and channels and changes in lagoon hydrology over the past 20 years [57,58]. Vast salina areas have become degraded as dyke erosion propagates to neighbouring salinas, which are often also abandoned when dyke reconstruction becomes too costly, and the surrounding vegetation, formerly controlled during salina maintenance, invaded the ponds, altering the characteristic landscape of the lagoon.

When salinas are abandoned, the lack of seasonal water level control and of dyke and pond maintenance also causes profound changes in fauna and flora and in the biological and ecological systems as a whole. With increasing water levels a valuable wetland habitat is lost, as areas become less favourable to the development of many aquatic species and feeding and nesting of waterbirds becomes more difficult [40]. The loss of salinas causes the disappearance of their characteristic ecological and biological systems [9,19,42]. Though, for waterbirds, even non-active jet preserved salinas are preferable abandoned ones [11].

The maintenance of the salina infrastructure, namely of the walls, sluices and channels, is essential to guaranty the sustainability of saline ecosystems and can be more easily guarantied if the salt production is maintained [19]. There is a potential for the rehabilitation of abandoned salinas, transforming them into active salinas with high biological value and economic activity [56]. Therefore, strategies have to be developed to make the traditional salt activity profitable while, concurrently, preserving the wetlands through conservation measures and a rational use.

From an economic point of view, it would be unrealistic to expect all salinas to produce salt again [59]. But even assuming that a large number of abandoned salt ponds will continue to exist, at variable states of degradation, (partial) salina rehabilitation should be promoted to at least create conditions that are similar to those that exist in (operating) salinas during the non-production season, i.e. when the salina is flooded. Next to structural reparation work, this would require cleaning and periodical monitoring of the system. Notice that, despite the loss of biodiversity in abandoned salinas, their ecological value remains high when they are well preserved, because of their potential as natural reserves.

## Alternative uses

### Tourism

To halt the decline of artisanal salt production in Portugal, incentives have to be given, creating better economic and financial conditions, as is already done in other EU countries [60]. Tourism is one of the most important, rapidly developing economic activities, especially considering the last half of the twentieth century, and is a potential alternative source of revenue for salinas [61].

Tourism generates income and will encourage salina maintenance, helping to preserve an ancient cultural tradition and a unique and biodiverse habitat. It will furthermore raise the awareness towards the conservation of their cultural and natural heritage, in locals and tourists. Salinas combine cultural and landscape values, and natural characteristics that allow coexistence of a great variability of organisms and high biodiversity [42]. The fact that the saline environment is fragile means however that not all forms of tourism are appropriate for them. The forms of tourism proposed for salinas, such as agrotourism, cultural, conference, maritime, gastronomic, and ecotourism (e.g. bird watching), are based on and should protect the unique characteristics and resources of these areas [61]. Tourism type and impact has to be controlled and compatible with environmental protection and artisanal salt production [42].

Salinas may be used for a combination of education, recreation and adventure activities. The region of Aveiro, for example, has an enormous potential for tourism and related activities. Recently, the local tourism sector is recognising that artisanal salt production constitutes an element that differentiates the region and that may become important again at a regional and also national level [42,62]. Like other salina regions in Portugal (e.g. Figueira da Foz), Aveiro has established a salt museum, in the Troncalhada salina, that seeks to provide knowledge and interpretation of the area. In addition to the actual production of traditional sea salt, it demonstrates the practice of salt-culture for educational, cultural and environmental purposes [14]. Salt museums may contribute to the preservation of the cultural heritage of an area and raise public awareness setting up environmental education projects [61].

Furthermore, a cluster of non-active yet well preserved salinas could be selected to function as an open-air life laboratory for the study of local fauna ecology and behaviour, and for educational purposes [11]. The species that occupy this niche are usually unique, and have many biochemical and physiological properties that have not been fully investigated [48]. Once these salinas are occupied, monitoring of the breeding populations will provide opportunities for long term studies of population dynamics in these important functional wetlands [63].

### Fish culture in Aveiro

Many abandoned salinas have been transformed into extensive or semi-intensive fish farming units (e.g. in Spain, Portugal, Italy and Greece). Current recoveries of salinas in Aveiro have been aimed at developing aquacultures only. Exceptions are two salinas owned by the University of Aveiro and the Troncalhada salina, belonging to the Municipality of Aveiro, which were recovered for the production of salt [9]. In the Ria de Aveiro and during the 80's and 90's, semi-intensive fish-cultures replaced salt businesses at a rate of 13 salinas per year. Reasons for this shift were the financial incentives given to aquaculture and the fact that fish culture offers a permanent activity (as opposed to the seasonal salt production) [42]. Seabass, seabream and eel have been the main species sustaining the semi-intensive fish culture activity in Aveiro [64]. So far, intensive aquaculture is not allowed in the Ria de Aveiro.

Alternative uses of salinas can be economically interesting, but they may cause damage to the environmental quality of the area [9]. Fish-culture on a large scale leads to the impoverishment of the salina landscape and to an aggravation of the conflicts with farmers. It also often causes adverse environmental impacts by reducing the emerged area available for birds and other species, increasing organic load and nutrient concentrations, diminishing dissolved oxygen levels and introducing hormones, antibiotics, pesticides and various compounds that affect the food chain. Exotic species can be introduced as well, causing ecological imbalances that affect feeding and nesting birds. The escape of introduced or genetically altered species can spread diseases, provoke unwanted hybridizations, etc. [65,66].

In the past 10 years, the number and production of fish culture units in Aveiro has decreased. This may be due to a combination of less or discontinued investment in infrastructures and of foreign competition, with more imported aquacultured fish, particularly seabass and seabream. Furthermore, in comparison to intensive fish culture, the semi-intensive systems required to protect the lagoon are less profitable. Nevertheless and although aquaculture constitutes an industrial activity with implicit risks for the environment, small-scale, controlled aquaculture that is well integrated into the environment may be a viable alternative to salt production in salinas [42]. Where fish-farms already exist, a management plan should consider actions to improve the habitat quality for birds. Abandoned salinas should be inventoried and environmental impact studies carried out to determine which salinas are ecologically the most important. Further analyses can be made to select the best salinas to recover as future nature reserves and those that can be transformed into fish-farms. If the land use plan allows further areas to be transformed into fish-farms, a management plan should be set up to define regulations, in order to mitigate negative impacts for the ecosystems right from the beginning [9,19,63].

### Combining salt production with the exploitation of other products

A salina can generate revenue in terms of salt production, producing a good quality food item, as well as through other products that can be marketed. The combination of salt production with other cultures in salinas may increase their efficiency and economic viability, avoiding further abandonment and destruction of irreplaceable biotopes [16,67]. For a rational use of the ecosystem as aquaculture and salt production unit, we need to know the system's ecological parameters, the biology and ecology of naturally resident species and the quantity of available (life) food. Aveiro salinas are likely to have adequate ecological conditions for the cultivation of certain naturally occurring mollusc, fish, shellfish and microalgae species with commercial value, including: the shore crab (*Carcinus maenas*), common cockle (*Cerastoderma edule*), grass shrimp (*Palaemonetes varians*), brine shrimp (*Artemia *sp.), seabream (*Sparus aurata*), seabass (*Dicentrarchus labrax*), Senegalese sole (*Solea senegalensis*), common eel (*Anguilla anguilla*), grey mullet (*Mugil cephalus*), European flounder (*Platichthys flesus*) and the green-algae *Dunaliella *sp.. These species occur naturally during the year or seasonally in salinas and survive without any artificial feeding [16,68].

During the 19^th ^century, fish culture in Aveiro salinas was used as an activity complementary to salt production, which took only place during the flooded, non-productive season (i.e. in the cold, rainy season). When the salina was emptied to prepare the new salt production season, the fish were captured and sold. For a better use of the potential of artisanal salinas, the combination of salt and fish culture could be extended to the salt production season, in the supply ponds. Therefore, a broader understanding of the population dynamics of ictiofauna in Aveiro salinas would be needed. To gain insight, a first step should include *in situ *observations, to identify species and their densities throughout the year, as well as their behaviour and resistance to increased salt levels. When the floodgates are closed, the lack of connections between the evaporators and crystallizers and other salina areas may cause significant mortality of fingerlings that may have entered during the non-production season.

As previously mentioned, a proper management of *Artemia *populations in salinas may optimize salt production in terms of both quality and quantity. But it can also constitute a valuable by-product, with commercialization of *Artemia *cysts and biomass. *Artemia *are used throughout the world as a source of live food for juvenile stages of commercial aquaculture species, e.g. in prawn and finfish hatcheries. The *Artemia *nauplii are considered as the best, sometimes the only, live feed for the larval stages of shrimp and fishes [67]. Studies on the biometric, chemical and hatching characteristics of the two existing *Artemia *strains in the Aveiro salinas (bisexual and parthenogenetic) allowed to conclude that they can be successfully used as larval food for several species of fish and shellfish. However, the hatching quality of these cysts can be improved with the employment of better collecting and processing techniques as well as better management practices of the salina ponds [16,69]. Cultivation of green algae *Dunaliella salina *and *D. bardawil *for the production of β-carotene is the major success story of halophile biotechnology [70]. The high carotenoid concentrations found in hypersaline microalgae are an advantage for aquaculture since they are necessary for pigmentation, vitamin activation, antioxidation, growth and possibly even reproduction of species [71]. Algae cultivation at salinas can also help achieve higher salinities that eventually assist higher production of salt [20]. Many other products synthesized by halophiles or processes performed by them have potential applications, but these have not yet led to commercially viable operations [70].

Although naturally occurring species suggest the highest probability of culture success, there is also the possibility of introducing other species, possibly of greater commercial value. Here we have to consider the risks to the ecosystem that may result from the introduction [27]. Creating the right conditions for introduced species may be easy, but confining them to a given number of ponds is more difficult, especially in an intricate saline ecosystem. Exotic species that escape and reproduce, becoming invasive species, would cause an imbalance and possible decline of the original aquatic flora and fauna of the water body. The long term effects may even result in the complete disappearance of native species, e.g. *Artemia *populations and other aquatic invertebrates, and a decline in the number of seabirds and shorebirds [63].

Only a preliminary environmental study can suggest which species can be cultivated, and what are the necessary work plans that need to be developed for their economic exploitation. Gained insights will suggest which changes should be applied to the system, e.g. biomanipulation, taking care not to damage the ecosystem. Well managed, a combined salt and other products culture may raise income considerably and make salinas biologically and economically more valuable.

## Overview

In the past decades the number of functioning salinas in Portugal has declined markedly, with serious negative consequences for the ecological equilibrium in these saline systems, losses of landscape quality and cultural heritage. To halt or invert this trend salinas have to become more profitable. Salt production and quality can be optimized, considering and managing the variable biological and physicochemical factors and applying biomanipulation if necessary. Alternative uses (e.g. fish culture) and production of profitable by-products (e.g. *Artemia *or algae) are options. And, salinas have an enormous potential for tourism and related activities, as they combine unique natural, cultural and historical values. These activities may generate revenues that allow a viable yet sensible exploitation of salinas, keeping their fundamental structural, physicochemical and biological characteristic, hence preserving these unique ecosystems and valuable habitats.

## Competing interests

The authors declare that they have no competing interests.

## Authors' contributions

CMR is responsible for the design and first draft of the manuscript. AB, FA and NV have carefully followed the writing of the said manuscript and substantially contributed for its improvement by exposing their critics, ideas and knowledge in the field of study. AB also assisted in polishing the English style. All authors read and approved the final manuscript.
